# Prophylactic Use of *Ganoderma lucidum* Extract May Inhibit *Mycobacterium tuberculosis* Replication in a New Mouse Model of Spontaneous Latent Tuberculosis Infection

**DOI:** 10.3389/fmicb.2015.01490

**Published:** 2016-01-08

**Authors:** Lingjun Zhan, Jun Tang, Shuzhu Lin, Yanfeng Xu, Yuhuan Xu, Chuan Qin

**Affiliations:** Key Laboratory of Human Diseases and Comparative Medicine, Ministry of Health, Institute of Laboratory Animal Science, Peking Union Medical College, Chinese Academy of Medical Sciences and Comparative Medicine CenterBeijing, China

**Keywords:** mouse model, spontaneous latent tuberculosis infection, *Ganoderma lucidum* extract, prophylactic use, inhibited *Mycobacterium tuberculosis* replication

## Abstract

A mouse model of spontaneous latent tuberculosis infection (LTBI) that mimics LTBI in humans is valuable for drug/vaccine development and the study of tuberculosis. However, most LTBI mouse models require interventions, and a spontaneous LTBI mouse model with a low bacterial load is difficult to establish. In this study, mice were IV-inoculated with 100 CFU *Mycobacterium tuberculosis* H37Rv, and a persistent LTBI was established with low bacterial loads (0.5~1.5log_10_ CFU in the lung; < 4log_10_ CFU in the spleen). Histopathological changes in the lung and spleen were mild during the first 20 weeks post-inoculation. The model was used to demonstrate the comparative effects of prophylactic and therapeutic administration of *Ganoderma lucidum* extract (spores and spores lipid) in preventing H37Rv replication in both lung and spleen. H37Rv was inhibited with prophylactic use of *G. lucidum* extract relative to that of the untreated control and therapy groups, and observed in the spleen and lung as early as post-inoculation week 3 and week 5 respectively. H37Rv infection in the therapy group was comparable to that of the untreated control mice. No significant mitigation of pathological changes was observed in either the prophylactic or therapeutic group. Our results suggest that this new LTBI mouse model is an efficient tool of testing anti-tuberculosis drug, the use of *G. lucidum* extract prior to *M. tuberculosis* infection may protect the host against bacterial replication to some extent.

## Introduction

Approximately one third of the world's population is infected with *Mycobacterium tuberculosis*, and about 90% of these infections are latent. A better understanding of mechanisms leading to latency is required to help prevent, control, treat, and eliminate tuberculosis infection and disease.

Similar to other latent infections, *M. tuberculosis* in latent tuberculosis infection (LTBI) replicates at low levels in the infected organs, and therefore histopathology results are absent or mild. There are several established LTBI animal models, including mice (Scanga et al., 1999), guinea pigs (Kashino et al., 2008), rats (Singhal et al., 2011), rabbits (Manabe et al., 2008), and nonhuman primates (Lin et al., 2009a). These models have been used to identify host factors that contribute to the establishment and maintenance of *M. tuberculosis* latency, and reactivation of replication. One of the most recognized LTBI mouse models is the Cornell model, which is created by inhibiting *M. tuberculosis* replication with intervening factors (Lenaerts et al., 2004; Woolhiser et al., 2007). However, there is no currently available mouse model of spontaneously paucibacillary tuberculosis.

The three primary parameters used for evaluating vaccine or drug candidate against tuberculosis are the bacterial load, pathological change in the lung, and the tuberculosis relapse rate in the latter phase of LTBI. With the current LTBI mouse model, the period of latency is relatively long, and to complete the evaluation can require 3–7 months (Ziv et al., 2001; Ha et al., 2003; Zhang et al., 2011). Thus, the current models are inefficient and costly, and slow the development of new tuberculosis vaccines and drug treatments.

A previous study showed that a proteoglycan extracted from the fruiting bodies of the bracket (polypore) fungus *G. lucidum* could be a preventative of diabetic complications (Pan et al., 2013). Furthermore, triterpenes of *G. lucidum* were shown to exert anti-lung cancer activity *in vitro* and *in vivo*, and such anti-cancer activity was mediated by enhancing immunomodulation and induction of cellular apoptosis (Feng et al., 2013). Also of note is that polysaccharides purified from the submerged culture of *G. formosanum* can activate macrophages and protect mice against *Listeria monocytogenes* infection (Wang et al., 2011). *G. lucidum* can also regulate natural killer cells (Chien et al., 2004), macrophages (Yeh et al., 2010), T cells (Lai et al., 2010; Yoshida et al., 2012), and dendritic cells (Meng et al., 2011), all of which are actively involved in the innate and adaptive immune responses to *M. tuberculosis* infection. Thus, we wondered whether *G. lucidum* could protect the host from *M. tuberculosis* infection.

To test our hypothesis, in the present study we established a novel mouse model of spontaneous LTBI, with a shorter latency period and lower bacterial load than previous models. We then evaluated the protective effects against *M. tuberculosis* infection in this new model exerted by the Takayama *G. Lucidum* (MeiShanTang, Hong Kong), which is rich in triterpenes, and is proven effective in treating simian acquired immune deficiency syndrome via the immune system (Lu et al., 2011).

## Materials and methods

### Ethics statement

The Institute of Animal Use and Care Committee of the Institute of Laboratory Animal Science, Peking Union Medical College approved all protocols and procedures that involved animals (ILAS-PC-2013-015). All mice were housed in plastic cages (6/cage) with free access to drinking water and a pellet diet, under controlled humidity (50 ± 10%), light (12/12 h light/dark cycle), and temperature (23 ± 2°C) conditions in an Animal Biosafety Level 3 (ABSL-3) facility. Prior to procedures performed at each time-point, mice were fasted overnight and then given anesthesia.

### Establishment of the LTBI mouse model

#### Bacterial strain

The *M. tuberculosis* strain H37Rv was first cultured in Löwenstein-Jensen plates (L-J plates) for 3 weeks, to mid-log phase. Then the bacteria were harvested using a sterilized L-shaped glass rod and resuspended in 0.9% NaCl in a glass grinder. The suspension was filtered through a 5 μm membrane and the bacterial density adjusted to ~1 × 10^7^ colony-forming units (CFU)/mL. An aliquot of bacilli were then plated on an L-J plate for CFU enumeration.

#### Mice

Specific-pathogen free female C57BL/6 mice (6–8 weeks old, *n* = 450) were obtained from Vital River Laboratory Animal Technology (China). Mice were maintained in an ABSL-3 specific pathogen-free facility and allowed food and water *ad libitum*.

#### Inoculation of mice with M. tuberculosis

The mice were randomly and equally apportioned to an infection or a blank control group, 150 mice for each experiment, 75 mice for infection and blank control group each, the experiment would be repeated 3 times with 1 week interval. Mice in the infection group were inoculated through the tail vein with 0.1 mL of H37Rv (1 × 10^3^ CFU/ mL), that was 100CFU for each mouse, and control mice were similarly injected with 0.1 mL 0.9% NaCl. All procedures were performed in a biosafety cabinet located in the ABSL-3 facility.

### Analysis of pathologic changes

Six mice were sacrificed at each time-point (weeks 1, 3, 5, 8, 12, 16, 20, and 24), for the first experiment, the rest mice were sacrificed at week 34, 38, 44 and 52(see Supplementary Image 2), six for each time-point, and for the latter two experiments, the rest mice were sacrificed at 52th week. The lung, spleen, and liver were removed from each animal; gross lesions were examined and described. The necropsy tissues were fixed and paraffin-embedded. The sections were cut and stained with hematoxylin and eosin, and reviewed by a veterinary pathologist. Semi-quantitative scores were assigned to reflect the lesion size and extent of inflammation of the entire lung field: +, 25%; ++, 50%; +++, 75%.

### Quantitative culture of lung and spleen homogenates

The lung and spleen tissues taken at necropsy were washed in 4% H_2_SO_4_ and homogenized in 0.9% NaCl. Serial dilutions of the homogenous lung fluid were used to inoculate the L-J medium culture in tubes, which were incubated at 37°C and 5% CO_2_. The bacterial counts in the tubes were read after 21 days. At each time-point, the mean of the bacterial loads in each organ type of 6 mice were calculated.

### Evaluation of the protective effect of *G. Lucidum* in the LTBI mouse model

#### Study design

The mice were divided into an LTBI mouse model control group, a *G. lucidum* prophylaxis group, and a therapy group. Mice in the control group were inoculated with *M. tuberculosis* as described above, but were fed normally and not given *G. lucidum* extract. The prophylaxis group received daily doses of *G. lucidum* mushroom extract (described below) beginning 1 month before *M. tuberculosis* inoculation and until 16 weeks after inoculation. In the therapy group, mice were given daily doses of *G. lucidum* mushroom extract beginning at the time of inoculation and lasting 16 weeks.

#### Preparation of *G. lucidum* extract-containing food

Takaya Shell-broken Ganoderma Lucid Spores and Spores Lipid, which were rich in terpene carbon dioxide extraction from included species of *G. lucidum* (MeiShanTang, Hong Kong) were mixed with the mouse feed powder and water, and then the mixture was manually formulated into ~15-cm-long feed strips with diameter of 1.5 cm. The strips were dried in the 37°C oven for 48 h. Each mouse received daily 15 mg of *G. lucidum* spores and 15 mg spore lipids in 4 g of feed (Lu et al., 2011).

#### Mouse trails

In the prophylaxis group, the feeding duration of *G. lucidum* was 20 weeks, starting 1 month before *M. tuberculosis* inoculation and continuing to the 16th week post-inoculation. In the therapy group, dietary *G. lucidum* (including spores and spore lipids) was administrated for 16 weeks, from the inoculation of *M. tuberculosis* to the 16th week post-inoculation. The effect of *G. lucidum* on *M. tuberculosis* infection in mice was evaluated according to the bacterial load in the lung and spleen, and the pathological changes in the lung, spleen, and liver at 3, 5, 8, and 16 weeks post-inoculation.

#### Fluorescence-activated cell sorting (FACS) analysis of immune cells

Dendritic, natural killer, CD4^+^/CD8^+^ T cells, and regulatory T (Treg) cells in the peripheral blood and lung were stained with specific antibody (all from eBioscience) for 30 min at 4°C. The following mouse antibodies were used: CD11b-fluorescein isothiocyanate (FITC) and CD4-FITC; I-AB-phycoerythrin (PE), CD86-PE, CD8-PE, CD49b-PE, and FOXP3-PE; CD80-peridinin chlorophyll protein complex (PERCP) and CD3-PERCP; and CD11c-allophycocyanin (APC), perforin-APC, and CD25-APC. The stained cells were analyzed by FACS.

### Statistical analyses

Quantitative data are expressed as the mean ± standard error of the mean (SEM), and analyzed using a two-tailed Student's *t*-test and ANOVA. *P* < 0.05 was considered statistically significant.

## Results

### Bacterial loads in the lung and spleen of the LTBI mouse

**Figure 1:**
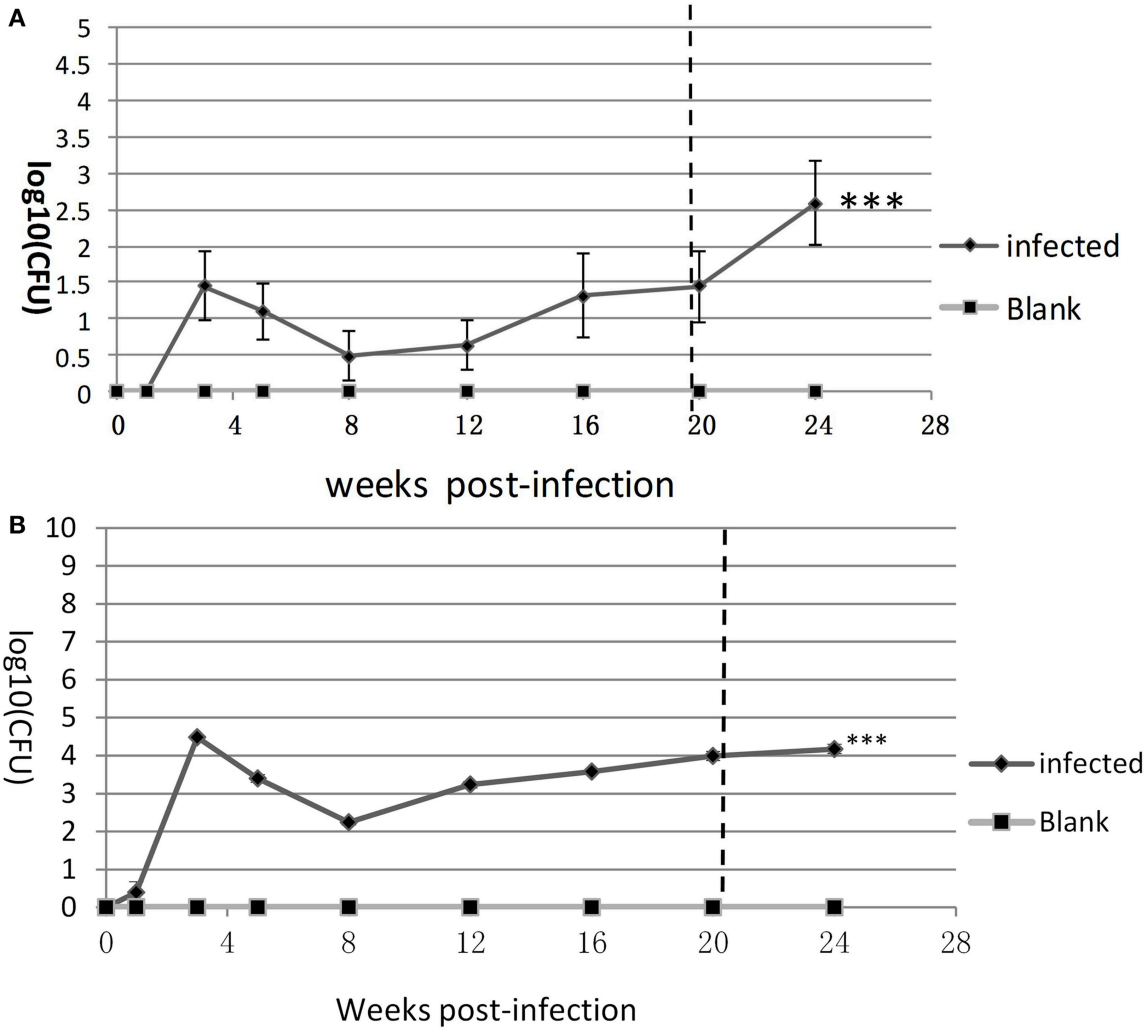
**The replication kinetics of ***M. tuberculosis*** in the lung and spleen**. Bacterial loads (log_10_CFU) in **(A)** the lung and **(B)** spleen were counted on the L-J medium from week 1 to week 24. Six mice for each time-point in each group. One of three similar experiments is shown. The SEMs are plotted as error bars. *T*-test and ANOVA analysis, ^***^P < 0.001.

The bacterial loads of lung and spleen in the infection and blank control groups at 1, 3, 5, 8, 12, 16, 20, and 24 weeks after inoculation with *M. tuberculosis* was determined (Figures 1A,B). No *M. tuberculosis* was detected in the blank control mice at any time-point. In the lungs of the infection group, the mean bacterial load was ~1.5log_10_ CFU at 3rd week, at the lowest level found (~0.5 log_10_ CFU) at 8th week, fluctuated from indeterminably low to ~2log_10_ CFU at 8–20th week, and was ~2.5 log_10_ CFU at week 24 (Figure 1A). In the spleens of the mice in the infection group, the mean bacterial load at 3rd week was 4.5log_10_ CFU, then 2.1log_10_ CFU at 8th week, and was subsequently progressively higher at each time-point, to >4 log_10_ CFU at week 24 (Figure 1B). Compared with blank control, both the bacteria loads of spleen and lung in infected group had significant statistic difference (^***^P < 0.001).

**Figure 2:**
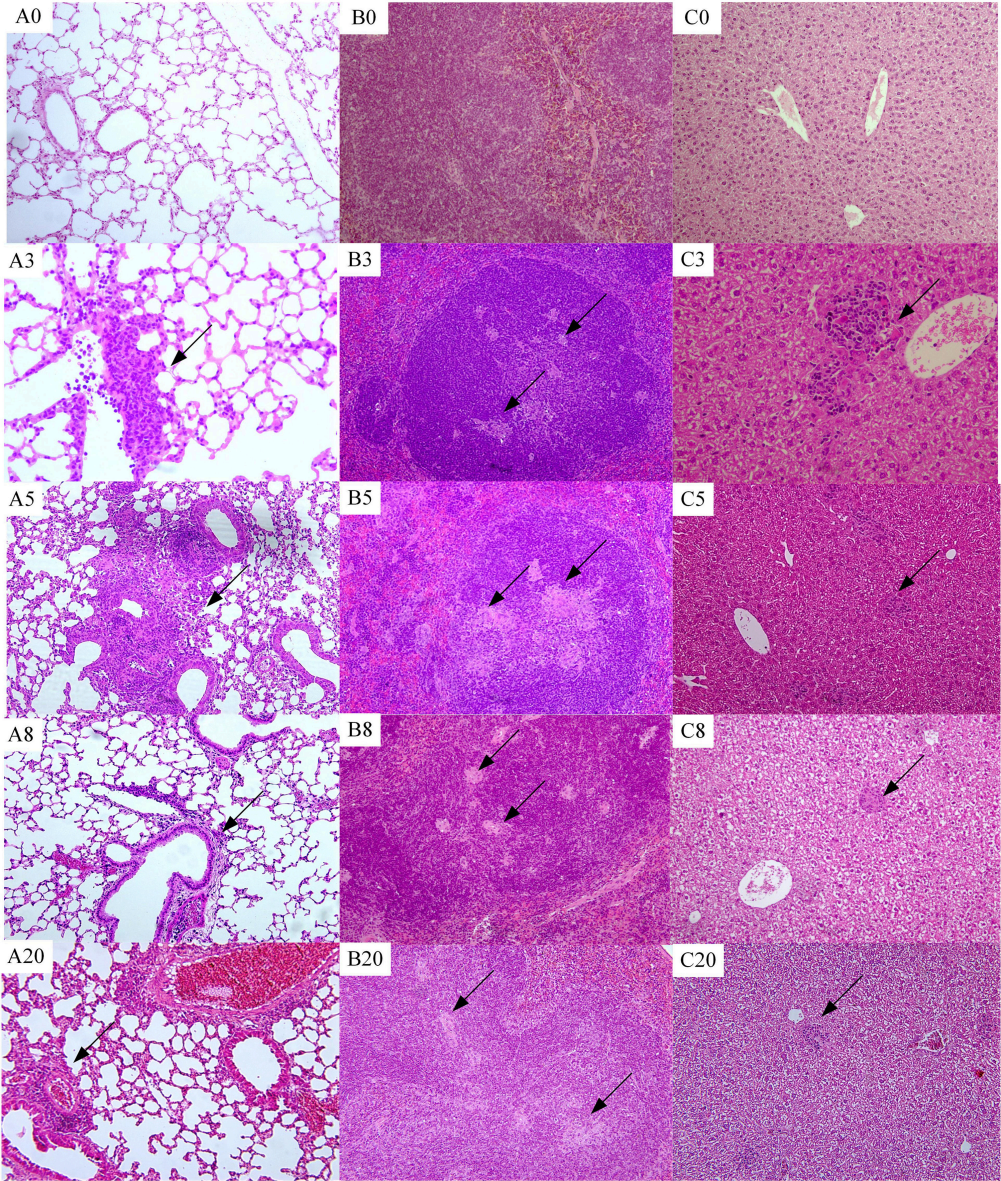
**Histopathological changes over time (100 ×)**. (A0–A20) Lung histology at weeks 0, 3, 5, 8, and 20, respectively. (B0–B20) Spleen histopathology at weeks 0, 3, 5, 8, and 20. (C0–C20) Liver histopathology at weeks 0, 3, 5, 8, and 20. Arrow indicates granulomas-like lesion.

There were no observed gross pathological changes throughout the infection course in any group, but histopathological changes were observed under the light microscope. In the lung at week 3, mild infiltration with inflammatory cells in the peripheral vascular was observed in the lung. At week 5, a granuloma-like structure in the lung was observed, and scored as a severe lesion (+++). However, at week 8, the histopathologic lesion was not seen, and only a few inflammatory cells infiltrated vessels. Infiltration of inflammatory cells in the lung was greater at week 16 (Figure 2).

In the spleen at 3rd week, several small granulomas (diameter, 5–50 μm) were detected in the white pulp. At week 5, the granulomas in the white pulp of the spleen were of diameters 20–150 μm, but at week 8 granuloma sizes were 5–20 μm. At week 20, several granuloma-like lesions of size 10–50 μm were observed in the spleen white pulp (Figure 2).

At week 3–16, granuloma-like lesions were occasionally observed in the hepatic lobule. The size of irregularly shaped granuloma-like lesions at week 3 was ~50 × 350 μm, and smaller granuloma-like lesions (20–50 μm) were present from weeks 5 to 20 (Figure 2).

### Bacterial loads in the lung and spleen of *G. lucidum* extract-treated mice

The condition of mice inoculated with 100 CFU *M. tuberculosis* resembled LTBI during the first 20 weeks post-inoculation. Therefore, to evaluate the effect of the *G. lucidum* extract, we determined the bacterial loads of the control and model mice at time-points up to 16 weeks post-inoculation.

**Figure 3:**
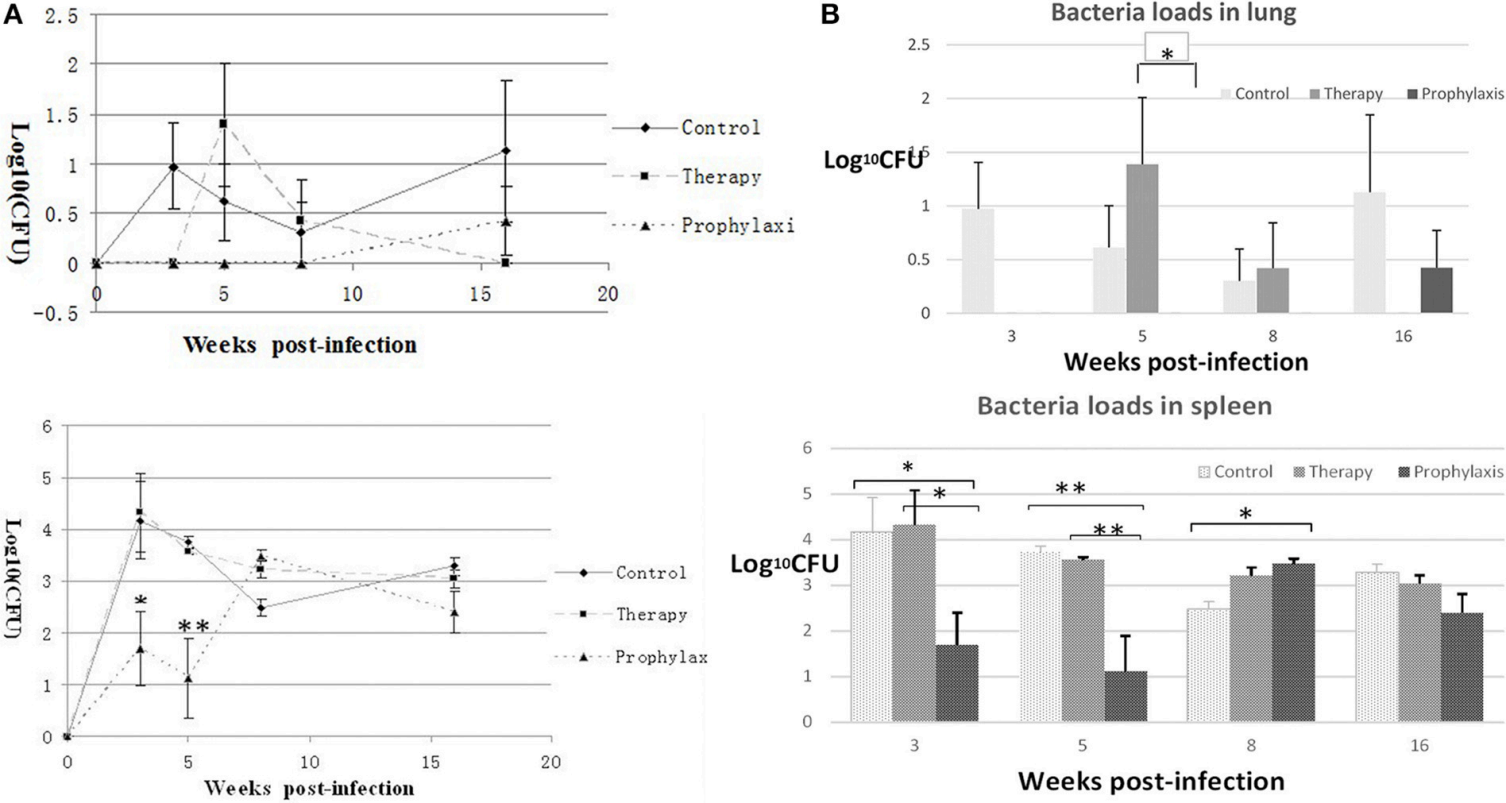
**Differences in bacterial loads between the control group and ***G. lucidum***-treated (therapy and prophylaxis) mice**. Bacterial loads (log_10_ CFU) in the **(A)** lung and **(B)** spleen were counted on the L-J medium from week 0 to week 16. One of three similar experiments is shown. The SEMs are plotted as error bars. ANOVA analysis, ^*^P < 0.05, ^**^P < 0.01.

In the prophylaxis group, in the lung the bacterial load was essentially none up through week 8, but was (0.42 ± 0.34) log_10_ CFU at week 16 (Figure 3A). In the therapy group, the *M. tuberculosis* level in the lung remained essentially undetectable through the first 3 weeks, but was (1.39 ± 0.77) log_10_ CFU at week 5, (0.42 ± 0.42) log_10_ CFU at week 8, and again undetectable at week 16. In the spleens of the prophylaxis group, the mean *M. tuberculosis* levels were (1.69 ± 0.71) log_10_ CFU at week 3, (1.12 ± 0.77) log_10_ CFU at week 5, (3.48 ± 0.11) log_10_ CFU at week 8, and (2.41 ± 0.41) log_10_ CFU at week 16. In the spleens of the therapy group, the bacterial loads were similar to that of the control group: a peak value at (4.33 ± 0.75) log_10_ CFU at week 3, (3.56 ± 0.05) log_10_ CFU at week 5, (3.22 ± 0.17) log_10_ CFU at week 8, and (3.04 ± 0.17) log_10_ CFU at week 16(Figure 3B). As for spleen bacteria loads, the prophylaxis group showed statistical differences at both week 3 and 5, compared with both blank control and therapy group (W3, *P* < 0.05; W5, *P* < 0.01), and only with control group at week 8(*P* < 0.05); but for lung bacteria loads, the prophylaxis group revealed significant statistic difference only at week 5, compared with therapy group (Figures 3A,B).

### Histopathologic changes in *G. lucidum*-treated mice

There were no macroscopic changes in the lungs, spleens, or livers in any of the groups, and no visible differences in the microscopic histology of the spleens or livers. However, histological differences in the lung were detected.

**Figure 4:**
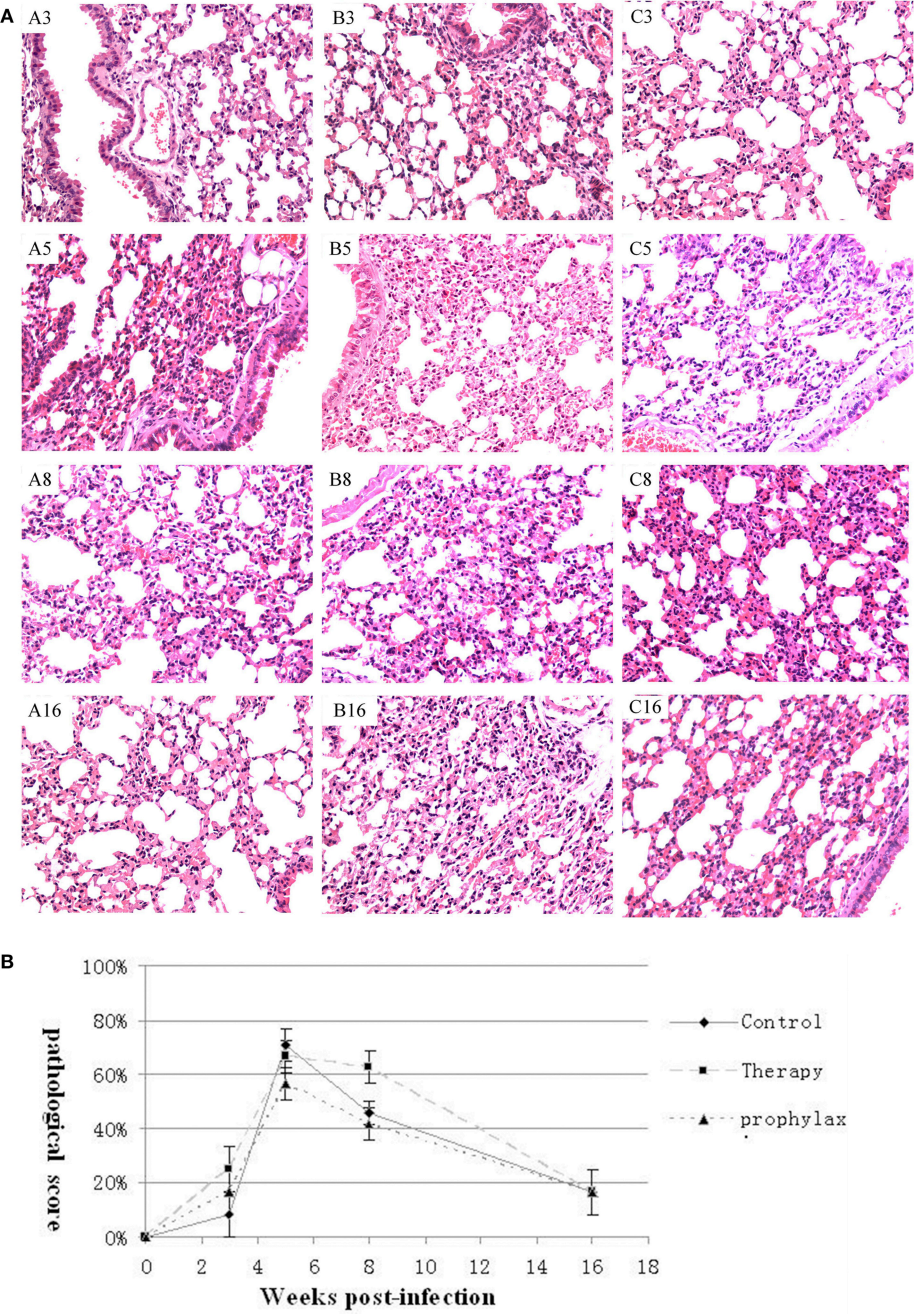
**Pathology of the lung. (A)** Histopathology of the lungs at weeks 3, 5, 8, and 16 weeks of representative mice in the (A3–A16) control, (B3–B16) prophylaxis, and (C3–C16) therapy groups (200 ×). **(B)** Pathological lesion scores in the lung of mice in the control, therapy and prophylaxis groups at weeks 3, 5, 8, and 16. SEMs are plotted (*P* > 0.05).

In the LTBI model control (untreated) group, inflammatory lesions were aggravated at week 5 compared with week 3, but were not seen from week 8 to 16. Similar to the control group, in the prophylaxis and therapy group small lesions peaked at week 5, and then were absent at week 8–16. At week 5, inflammation in the prophylaxis group was milder than inflammation in the control group (Figure 4A). Compared with the control group, mice in the *G. lucidum* prophylaxis group had lower pathology scores during the infection course, but the difference was not significant (*P* > 0.05; Figure 4B).

### Percentages of dendritic cell, natural killer cell, and treg cell, and the ratios of CD4^+^/CD8^+^T cell in blood

**Figure 5:**
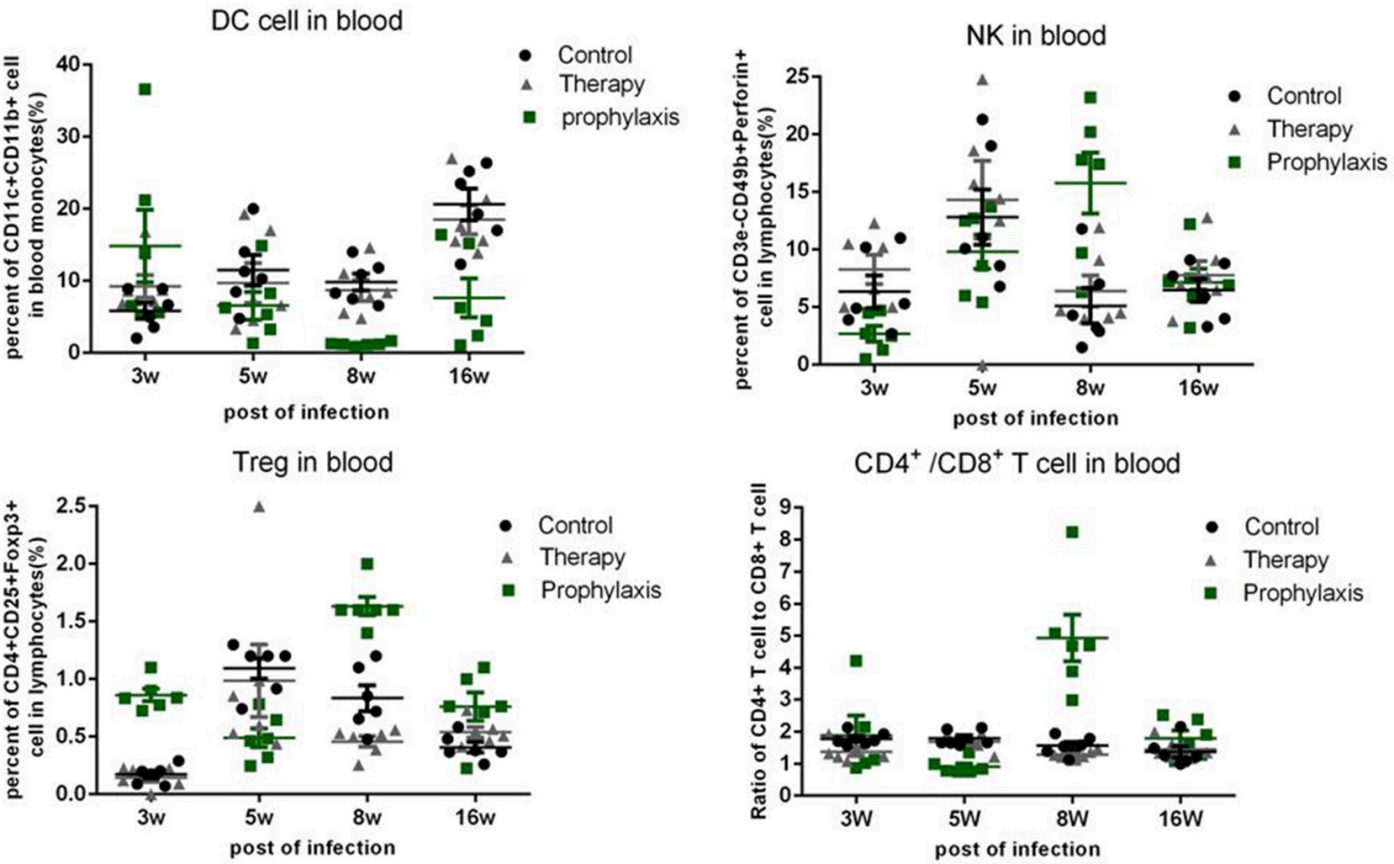
**Percentages of dendritic cells (DC), natural killer (NK) cells, and Treg cells, and the ratios of CD4^**+**^/CD8^**+**^T cells in the blood**. The results are expressed as mean ± SEM, plotted using GraphPad Prism version 4.01 (GraphPad, San Diego, CA).

The number of dendritic cell, natural killer cell, Treg cell, and the ratio of CD4^+^/CD8^+^ T cell in the peripheral blood were analyzed by FACS (Figure 5). In the prophylaxis group, the percentages of dendritic cell were 14.8 ± 5.04% at week 3, 6.56 ± 1.93% at week 5, 1.22 ± 0.10% at week 8, and 7.64 ± 2.69% at week 16. In the therapy and control group, the percentages of dendritic cells were similar to that of the prophylaxis group, progressively increasing over time from 9.24 ± 1.55% at week 3 to 18.48 ± 2.00% at week 16.

The percentages of natural killer cell in the prophylaxis group was 2.70 ± 0.68% at week 3, 9.82 ± 1.48% at week 5, 15.76 ± 2.63% at week 8, and 7.15 ± 1.20% at week 16. The percentages of natural killer cell in the control and therapy group were comparable: ~2, ~10, ~5, and ~7% at weeks 3, 5, 8, and 16, respectively.

The ratios of CD4^+^/CD8^+^T cells in all three groups were similar and stable at ~1.8 throughout the infection period, except for the prophylaxis group, in which a mean ratio of ~4.2 was observed at week 8.

The percentages of Treg cells in the prophylaxis group were 0.86 ± 0.05% at week 3, 0.49 ± 0.08% at week 5, 1.63 ± 0.08% at week 8, and 0.76 ± 0.12% at week 16. In the control and therapy group, the percentages of Treg cells were similar (~1%) at week 5, and progressively and similarly decreased from week 5 to16 (Figure 5).

## Discussion

We present here a novel mouse model of spontaneous LTBI, which closely resembles human LTBI. The model was generated through experimental inoculation of *M. tuberculosis* without resorting to other means such as anti-tuberculosis drugs treatment or *Mycobacterium bovis* Bacillus Calmette-Guerin vaccination. A useful application of this model is to evaluate anti-tuberculosis drugs or vaccines during the early latency phase, which would be more efficient than the previous models (Ziv et al., 2001; Ha et al., 2003; Zhang et al., 2011). In the present study, we demonstrated the efficiency of the new mouse LTBI model by testing the effect of *G. lucidum* extract on *M. tuberculosis* infection, by inhibiting *M. tuberculosis* replication.

To initiate the LTBI model, C57BL/6 mice were IV-injected with 100 CFU *M. tuberculosis* inoculum. *M. Tuberculosis* initially replicated in the lung and spleen, with bacterial loads rising until week 3, after which infection entered into a latent phase with *M. tuberculosis* levels declining from week 3 and stabilizing at a lower level till week 20. In accord with the bacterial load findings, pathological changes were detected at week 3, which became prominent at week 5, and then subsided to only mild perivascular inflammation from week 8 to 20. *M. tuberculosis* replication shifted from relatively active to dormant, resulting in lower bacterial loads and mild pathological changes in both the lung and spleen from weeks 8 to 20 post-inoculation. Thus, the period within 8 weeks would be regarded as “pre-latency phase,” and the period from week 8 to week 20 comprised the real latency phase of *M. tuberculosis* infection in this model (Figures 2, 3). The bacteria load curves of spleen and lung were similar to others study, but with lower value and shorter latency period, which would be more efficient in drug evaluation.

Treatment of mice with *G. lucidum* extract beginning 1 month before inoculation with *M. tuberculosis* (the prophylaxis group) inhibited replication of the bacterium in the lung and spleen during the first 8 weeks post-inoculation, relative to untreated control mice and mice treated from the time of inoculation (the therapy group). This inhibition was especially evident in the spleen 5 weeks after inoculation. No significant inhibition of *M. tuberculosis* replication was observed in the therapy group, suggesting that early use of *G. lucidum* extract is probably required for exerting anti- *tuberculosis* activity in this mouse model (Figure 4).

The changes of immune cell percentages in the peripheral blood did not correlate with the bacterial loads of *M. tuberculosis* or pathological changes observed in the lung and spleen. However, responses of the peripheral blood and lung to inoculation were opposed, with regard to percentages of dendritic cells. At post-inoculation week 3, the number of peripheral dendritic cells in prophylaxis group was higher than that in the control group, but at week 5 was lower than the control group, while the number of dendritic cells in the prophylaxis group lung was higher at week 5 than week 3 (Supplementary Data), and higher than the control group. These changes suggest that dendritic cells may have transferred from the peripheral blood to the lung from week 3 to week 5, and the accumulation of dendritic cells in the lung might participate in and augment the repression of *M. tuberculosis* replication, inferred from the immune effect of *G. lucidum* on dendritic cells in previous research (Figure 5) (Lin et al., 2009b; Meng et al., 2011).

However, the immune mechanism of *G. lucidum* extract could not be discerned, and nor if the effective constituent was terpenes, which should be investigated in a further study.

In conclusion, we established a mouse model resembling human *M. tuberculosis* LTBI. The early acute phase of infection (from inoculation to week 8) and the relatively short latency period makes this model useful for evaluating anti-tuberculosis drugs. We utilized the model to investigate the effect of *G. lucidum* extract on *M. tuberculosis* infection. We found that administration of Takaya *G. lucidum* extract prior to inoculation of *M. tuberculosis* was associated with lower levels of bacterial replication in this model.

## Author contributions

LZ and CQ conceived the concept and designed the experiment. LZ, JT, and SL conducted all the experiments except for the pathological analysis. YX and YX performed the pathology examinations. YL provided the Takaya *Ganoderma lucidum* extract. LZ performed the data analysis and wrote the manuscript. All the authors read and approved this manuscript for publication.

### Conflict of interest statement

The authors declare that the research was conducted in the absence of any commercial or financial relationships that could be construed as a potential conflict of interest.
