# Supplementary figures and images for: Prophylactic Use of Ganoderma lucidum Extract May Inhibit Mycobacterium tuberculosis Replication in a New Mouse Model of Spontaneous Latent Tuberculosis Infection
Source: Front Microbiol. 2016 Jan 8;6:1490. doi: 10.3389/fmicb.2015.01490 (PMC4705449; doi:10.3389/fmicb.2015.01490)

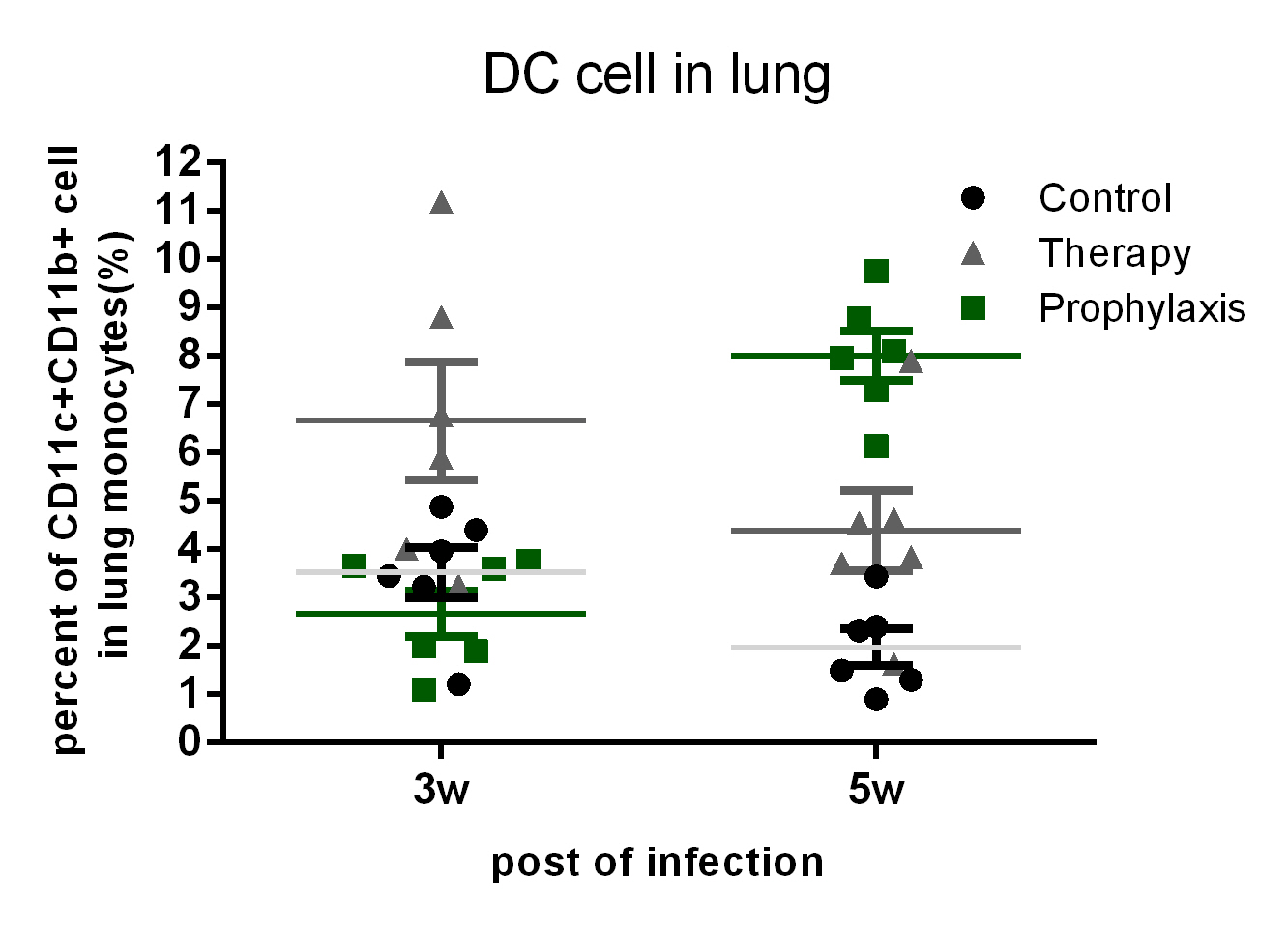

Supplement: Supplementary file 1 [file Image1.JPEG]
