# Supplementary material for: Prophylactic Use of Ganoderma lucidum Extract May Inhibit Mycobacterium tuberculosis Replication in a New Mouse Model of Spontaneous Latent Tuberculosis Infection
Source: Front Microbiol. 2016 Jan 8;6:1490. doi: 10.3389/fmicb.2015.01490 (PMC4705449; doi:10.3389/fmicb.2015.01490)
Supplement: Supplementary file 2 [file Image2.PDF]

Bacteria load in lung and spleen in latent tuberculosis infection model

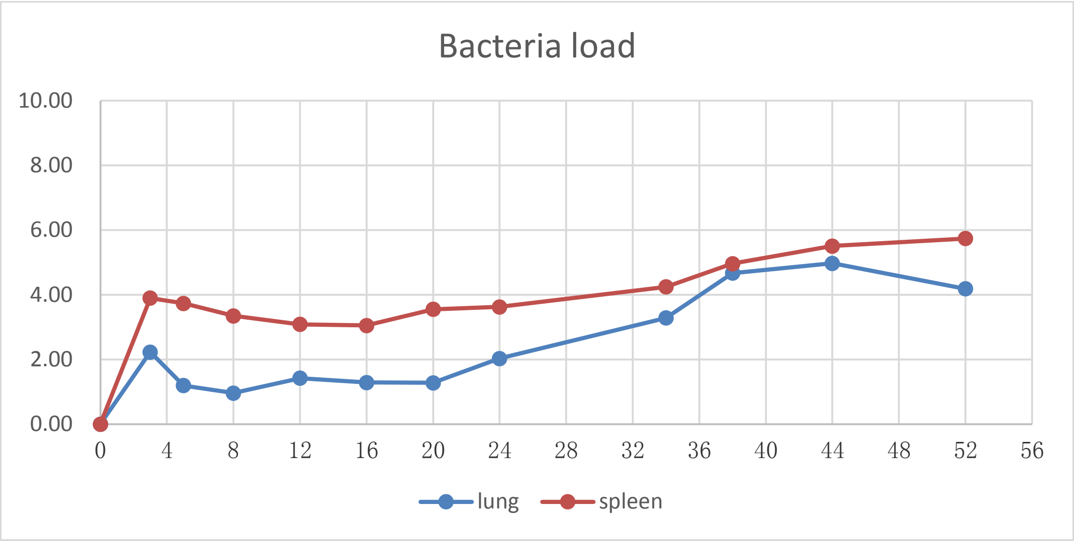

X axis: weeks postinfection

Y axis: Log10(CFU)
